# The effectiveness and cost-effectiveness of the Incredible Years^®^ Teacher Classroom Management programme in primary school children: results of the STARS cluster randomised controlled trial

**DOI:** 10.1017/S0033291718001484

**Published:** 2018-07-18

**Authors:** Tamsin Ford, Rachel Hayes, Sarah Byford, Vanessa Edwards, Malcolm Fletcher, Stuart Logan, Brahm Norwich, Will Pritchard, Kate Allen, Matthew Allwood, Poushali Ganguli, Katie Grimes, Lorraine Hansford, Bryony Longdon, Shelley Norman, Anna Price, Obioha C. Ukoumunne

**Affiliations:** 1 University of Exeter Medical School, South Cloisters, St Luke's Campus, Exeter, EX1 2LU, UK; 2 King's College London, King's Health Economics, Box PO24, Institute of Psychiatry, Psychology & Neuroscience, De Crespigny Park, London, SE5 8AF, UK; 3 Graduate School of Education, University of Exeter, North Cloisters, St Luke's Campus, Exeter, EX1 2LU, UK; 4 Education and Early Years, Cornwall County Council, 3 West, New County Hall, Treyew Road, Truro, TR1 3AY Truro, TR1 3AY, UK; 5 Educational and Counselling Psychology and Special Education, University of British Columbia, 2125 Main Mall, Vancouver, British Columbia, Canada, V6T 1Z4, Canada; 6 University of Exeter, Sir Henry Wellcome Building, Streatham campus, University of Exeter, EX4 4QG, UK; 7 NIHR CLAHRC South West Peninsula (PenCLAHRC), University of Exeter, South Cloisters, St Luke's Campus, Exeter, EX1 2LU, UK

## Abstract

**Background:**

We evaluated the effectiveness and cost-effectiveness of the Incredible Years^®^ Teacher Classroom Management (TCM) programme as a universal intervention, given schools’ important influence on child mental health.

**Methods:**

A two-arm, pragmatic, parallel group, superiority, cluster randomised controlled trial recruited three cohorts of schools (clusters) between 2012 and 2014, randomising them to TCM (intervention) or Teaching As Usual (TAU-control). TCM was delivered to teachers in six whole-day sessions, spread over 6 months. Schools and teachers were not masked to allocation. The primary outcome was teacher-reported Strengths and Difficulties Questionnaire (SDQ) Total Difficulties score. Random effects linear regression and marginal logistic regression models using Generalised Estimating Equations were used to analyse the outcomes. Trial registration: ISRCTN84130388.

**Results:**

Eighty schools (2075 children) were enrolled; 40 (1037 children) to TCM and 40 (1038 children) to TAU. Outcome data were collected at 9, 18, and 30-months for 96, 89, and 85% of children, respectively. The intervention reduced the SDQ-Total Difficulties score at 9 months (mean (s.d.):5.5 (5.4) in TCM *v.* 6.2 (6.2) in TAU; adjusted mean difference = −1.0; 95% CI−1.9 to −0.1; *p* = 0.03) but this did not persist at 18 or 30 months. Cost-effectiveness analysis suggested that TCM may be cost-effective compared with TAU at 30-months, but this result was associated with uncertainty so no firm conclusions can be drawn. A priori subgroup analyses suggested TCM is more effective for children with poor mental health.

**Conclusions:**

TCM provided a small, short-term improvement to children's mental health particularly for children who are already struggling.

## Introduction

Poor childhood mental health is common, persistent, and associated with many adverse outcomes (Costello and Maughan, 2015; Ford *et al.*, 2017). As three quarters of adults with poor mental health first experience difficulties in childhood (Kim-Cohen *et al.*, 2003), and parents and children are more likely to first contact teachers and specialist education professionals about mental health concerns than their GP (generalised practitioner), paediatricians or specialist child and adolescent mental health services (Ford *et al.*, 2007), there is a growing policy focus on children's mental health and the role of schools in particular (Department of Health and Department of Education, 2017).

Several overlapping populations of children can be conceptualised as having poor mental health (Wolpert, 2009) and confusion arises from the use of different terms to describe these children by practitioners from different disciplines. For example, educators discuss socio-emotional and behaviour difficulties, which describes a similar but not identical population of children who are considered to have psychopathology or mental health conditions by health-based professionals. In the current paper, the language applied reflects the literature quoted for these overlapping constructs.

The commonest type of childhood mental health problem is conduct disorder, a psychiatric diagnosis used to describe behaviour that challenges social norms to the extent that the child's ability to function is adversely effected (Ford *et al.*, 2017). Commonly comorbid with other mental health problems, conduct disorder affects 5–8% of the school-age population (Costello *et al.*, 2005), and predicts to all types of adult mental disorder (Kim-Cohen *et al.*, 2003). It is the most persistent of the common childhood disorders (Ford *et al.*, 2017), while the impairment and societal costs are not only confined to those with the most severe difficulties (Scott *et al.*, 2001). Affected children incur substantial costs to society and their families (Scott *et al.*, 2001). Although parent training courses are effective interventions to improve difficulties at home (Leijten *et al.*, 2018), they rarely improve school-based behavioural problems (Scott *et al.*, 2010). Children living in deprived circumstances are at greater risk than their more affluent peers of poor mental health in general and conduct disorder in particular (Ford *et al.*, 2004). They also often lack the socio-emotional competencies to thrive at school, while their parents face multiple barriers to attendance at parenting programmes (McEvoy and Welker, 2000; Ford *et al.*, 2004). A school-based alternative to parent training might reduce mental health inequalities and reach children whose difficulties persist despite treatment.

For each child who meets diagnostic criteria, there are probably three or four others with poor mental health, as when psychological distress is measured using a dimensional approach, there is a continuous spectrum of psychological functioning (Goodman and Goodman, 2011; Ford and Parker, 2016). The level of impairment for any given constellation of difficulties will be influenced by the child's social and psychological context, of which school is an important component. Effective school-based public mental health interventions could potentially improve functioning across the whole population as well as among children currently experiencing difficulties (Huppert and So, 2013).

Teachers complain of inadequate training to manage socio-emotional difficulties and challenging behaviour, and these difficulties are associated with higher stress levels, poorer mental health, burnout, and exit from the profession (Webster-Stratton *et al.*, 2001; Jennings and Greenberg, 2009). Disruption in the classroom can undermine the quality of teaching and interrupt the learning of all the children in the class (Jenkins and Ueno, 2017). An intervention that supports teachers to optimise children's mental health and behaviour might benefit every child subsequently taught by that teacher as well as the teacher themselves, and might be substantially more cost-effective than direct work with successive cohorts of children.

The Incredible Years^®^ Teacher Classroom Management (TCM) course has been identified by a recent systematic review of interventions that aim to improve children's mental health through training teachers as the school-based programme with the most evidence (Whear *et al.*, 2013). TCM draws on theories of how coercive cycles of interaction between adults and children reinforce the disruptive behaviour (Patterson, 1982); the importance of modelling and self-efficacy (Bandura, 1977); and developmental interactive learning methods (Piaget and Inhelder, 1962). It also incorporates cognitive behavioural approaches and Bowlby's attachment theory on the importance of positive relationships (Bowlby, 1951). Few previous studies have examined TCM without the parallel parent or child programmes and most have studied outcomes only for children with poor mental health and/or added additional coaching for teachers (Raver *et al.*, 2008; Webster-Stratton *et al.*, 2008; Baker-Henningham *et al.*, 2009; Kirkhaug *et al.*, 2016; Webster-Stratton, 2016; Hickey *et al.*, 2017). At the time the STARS (Supporting Teachers and childRen in Schools) trial began, only two small randomised trials of TCM in isolation from other interventions had been completed. Both suggested that TCM changed teachers’ behaviour but lacked the power to detect any impact on child mental health and did not evaluate cost-effectiveness (Martin, 2009; Hutchings *et al.*, 2013; Hickey *et al.*, 2017). Two other studies suggest that TCM improved the mental health of pre-school children, but included additional components with the TCM course (Baker-Henningham *et al.*, 2012; Fossum *et al.*, 2017). One recently completed trial in North Carolina detected a positive impact on school climate but did not detect an effect on child outcomes (Murray *et al.*, 2018). A priori subgroup analysis in this most recent trial suggested that children in poor initial mental health did benefit from exposure to teachers who attended the course. The STARS trial is to our knowledge the first large UK-based randomised trial with sufficient power to detect the impact of TCM in isolation from other interventions among primary school-aged children.

We evaluated whether TCM improved children's mental health (primary outcome), enjoyment of school and behaviour, and if so, whether any impact was sustained and whether TCM was cost-effective.

## Methods

The trial was conducted and reported in accordance with CONSORT and TIDieR guidelines (Schulz *et al.*, 2010; Campbell *et al.*, 2012; Hoffmann *et al.*, 2014). The study design and procedures are presented in full in the published trial protocol (Ford *et al.*, 2012) which was approved by the Trial Steering Committee (TSC) and Data Monitoring Committee (DMC). Ethical approval for the conduct of the trial was obtained from the Peninsula College of Medicine and Dentistry Research Ethics Committee (12/03/141).

### Study design and participants

STARS was a multi-centre, two-arm, pragmatic, parallel group, superiority, cluster randomised controlled trial designed to evaluate whether the Incredible Years^®^ TCM course (delivered at class level) improved the mental health of individual children. Children aged between 4 and 9 years (Reception to Year 4) were recruited and followed up after 9, 18, and 30-months. In keeping with previous studies, and the aims of TCM to promote socio-emotional regulation as well as improved behaviour, the primary outcome was the teacher-completed Strengths and Difficulties Questionnaire (SDQ) Total Difficulties score (Goodman, 2001). Schools (clusters) were allocated to TCM training (intervention) or teaching as usual (TAU; control). One class (teacher and all pupils) was selected by the headteacher from each school independently of the research team. Cluster randomisation was necessary because TCM provides teachers with skills that are applied to the whole class.

Schools across the South West of England were recruited in three cohorts for baseline data collection in September 2012 (Cohort 1), September 2013 (Cohort 2), and September 2014 (Cohort 3); each school could only participate in one cohort. Schools were approached through unsolicited contact with headteachers and publicity at local conferences. To be eligible for inclusion, schools needed a single-year class with 15 or more children aged between 4 and 9 years, taught by a teacher who held classroom responsibility for at least 4 days per week. Schools were excluded if they primarily taught pupils with special educational needs, lacked a substantive headteacher, or were judged as failing in their last Ofsted inspection (Office for Standards in Education, Children's Services and Skills; the official inspectorate for schools in England). All children in the selected classes were eligible for inclusion provided the class teacher judged that they and their parents had sufficient English language comprehension to understand recruitment information and complete outcome measures.

Written consent was obtained from the headteacher for the school's participation and from the class teacher for their involvement after nomination by the headteacher. Parents could ‘opt-out’ their child from the research and verbal assent was obtained from children each time they were asked to complete a questionnaire.

### Randomisation and masking

Randomisation of schools was completed after baseline data collection to avoid recruitment and response bias (Eldridge *et al.*, 2009). An independent researcher based at the University of Exeter who was masked to the identity of the schools to ensure allocation concealment computer-generated random numbers. The allocation was passed to the trial manager who then informed the schools. The allocation was completed separately for each cohort with all schools in that cohort allocated en block. An equal number of schools were allocated to each arm overall, but unequal allocation ratios to trial and intervention arms were necessary for Cohorts 1 and 3 to optimise the number of teachers available for each TCM training groups.

English primary schools cover two ‘Key Stages’ of education; Key Stage 1 (Reception to Year 2 or children aged 4–7 years) and Key State 2 (Years 3–6 or children aged 7–11 years). Only children in Key Stage 1 and Years 3 and 4 from Key Stage 2 were included in the STARS trial. The allocation was balanced on the following school factors: urban *v.* rural/semi-rural area; Key Stage 1 *v.* Key Stage 2); and deprivation [whether the % of children eligible for free school meals was greater than 19%, the UK national average in 2012 (Department of Education)]. We randomly generated one million potential allocations for each cohort and randomly selected the chosen allocation sequence from the 5% with the least imbalance on the above school factors (Raab and Butcher, 2001).

We were unable to mask the schools and teachers since the school needed to release the class teacher to attend the training. Children and parents were not informed whether their teacher attended training. The main research team were not masked as feasibility work indicated that visual cues in the classroom and enthusiastic comments from teachers would undermine attempts to do so, however, teachers and parents completed their measures independently of researchers, as did children aged 7 plus.

### Procedures and intervention

**Fig. 1.**
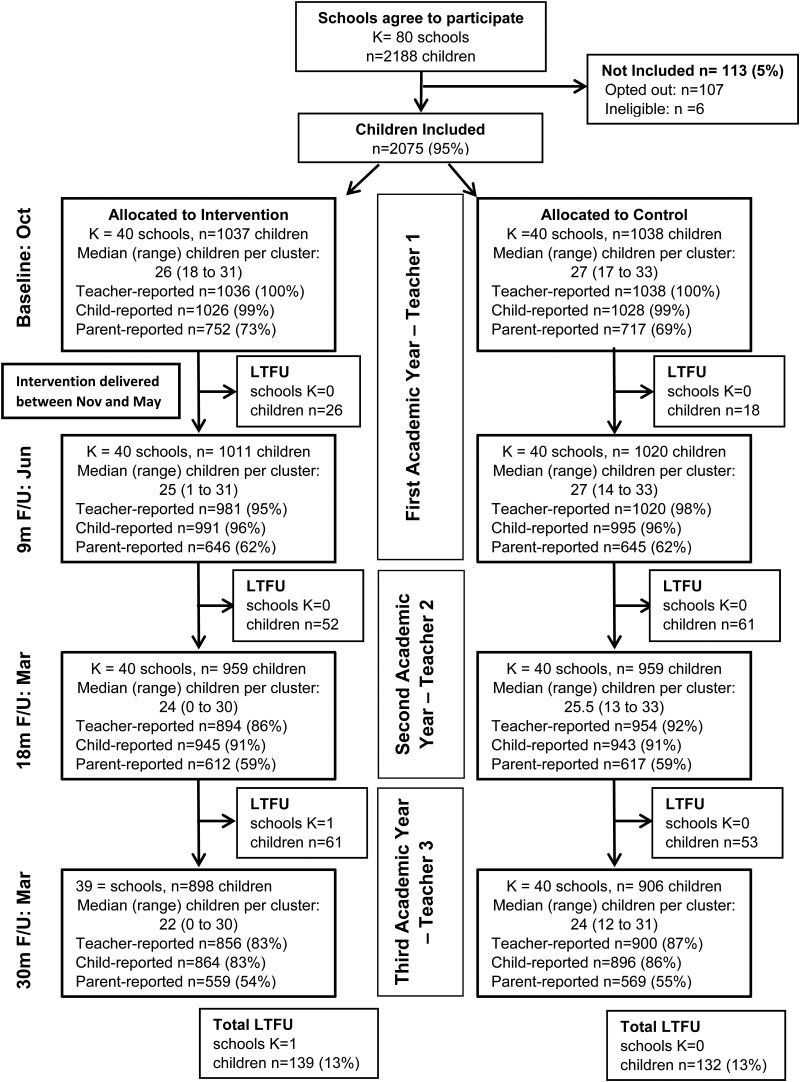
CONSORT diagram to illustrate data completeness related to the primary outcome.

TCM was delivered to groups of teachers in six whole-day sessions spread between October and June in the first academic year of each school's participation in the study after the collection of baseline measures and randomisation (see Fig. 1). The sessions took place during the school day but at an external venue. The facilitating group leaders were behaviour support practitioners and delivered in pairs. They completed mandatory TCM basic training, and led at least two previous courses. They received monthly supervision from the programme developers, which included video reviews of each session, to ensure fidelity to the model.

TCM is highly manualised with clear criteria for training, supervision, and fidelity, but allows ‘adaptation with fidelity’ in that group leaders can select from a range of techniques to deliver the prescribed curriculum in the manner most acceptable to their context (Webster-Stratton *et al.*, 2011). TCM's explicit goals are to: enhance teacher classroom management skills and improve teacher–student relationships; assist teachers to develop effective proactive behaviour plans; encourage teachers to adopt and promote emotional regulation skills; and encourage teachers to strengthen positive teacher–parent relationships. This is accomplished through goal-setting, reflective learning, video-modelling, and role play, with cognitive and emotional self-regulation training. Teachers were encouraged to practice novel strategies between sessions and discuss their experiences.

As recommended by the education community and to incentivise recruitment and retention, TAU schools were offered TCM training during their second year of involvement in STARS as long as the attending teacher did not teach the study children during the study follow-up. Schools access to other training and support services was not restricted.

### Outcomes

Teachers and parents completed the Strengths and Difficulties Questionnaire (SDQ) (Goodman, 2001), which is a widely used measure of mental health in childhood that includes 25 items (each scored 0–2) comprised five subscales, each with five items. The SDQ Total Difficulties score sums the *Behaviour*, *Emotions*, *Inattention/Overactivity*, and *Peer relationships* subscales with possible scores that range from 0 to 40. Higher scores indicate poorer mental health on all except for the Pro-Social subscale, where higher scores indicate better Pro-social skills. The SDQ *Impact* supplement is comprised of three items for teachers (possible score of 0–9) or five items for parents (0–15) and quantifies the extent to which difficulties in the areas of emotions, concentration, behaviour, or being able to get on with other people impact on the child's everyday life in terms of peer relations and classroom learning for both informants, and family life and leisure activities in addition for parents.

Our primary outcome was the SDQ-Total Difficulties completed by the children's class teacher. We also analysed binary versions of the SDQ-Total Difficulties score, with children scoring above the 80th centile for the British school-age population classified as *struggling*; (scored ⩾12 on teacher report or ⩾14 out of 40 on parent report) (Goodman, 2015) and the Impact score (scored one or more indicating some degree of *impairment*), as well as the subscales totals, for each informant.

Teachers also completed the Pupil Behaviour Questionnaire (PBQ: Allwood *et al.*, 2018) which measures the low level classroom-based disruptive behaviours commonly displayed by primary school-aged children. It includes six questions each scored: 0 = never, 1 = occasionally, 2 = frequently. Items are summed with a higher total score (possible range: 0–12) indicating more disruptive behaviour.

Parents also completed a brief, self-report version of the Child and Adolescent Service Use Schedule (CA-SUS) (Byford *et al.*, 1999; Harrington *et al.*, 2000; Barrett *et al.*, 2006) that comprised 13 items to collect data on children's use of key services (high cost, high frequency of use) of relevance to this population (see online Supplementary material). Teachers and parents reported demographic characteristics.

Children completed the *How I Feel About My School* measure (HIFAMS: Allen *et al.*, 2017) which measures children's attitudes towards school; possible scores range from 0 to 14 (summed across seven items each scored from 0 to 2), with higher scores indicating greater happiness at school.

Baseline and 9-month assessments took place during the first academic year of each school's participation, before and after the intervention was delivered respectively, and so were completed by the same teacher. The 18-month and 30-month assessments were completed by two different teachers as they were collected in the subsequent academic years (see Fig. 1). To enable a supply teacher to supervise the teacher's class, schools received £80 for each time-point that teachers completed the outcomes (£320 in total) and £160 for each training day attended (£960 in total). Teachers received a £10 gift voucher after outcome completion at each timepoint. Parent questionnaires were completed simultaneously to the teacher measures at baseline, 9, 18, and 30 months and were sent home via participating children. Parents received reminders via the school office and where possible second questionnaires were mailed directly to the home. Parents received a £5 gift voucher for every completed questionnaire. Child-reported outcome data were collected during school time by researchers as a classroom activity for children aged 7 or more, and individually for younger children. School staff were present but were instructed not to assist the children who were offered stickers at the end of each data collection.

### Statistical analysis

We assumed that each class would contain 30 children, 21 (70%) of whom would participate and 19 would be retained (90% of participants). With an intra-cluster (intra-school) correlation coefficient (ICC) of 0.15, based on data from Sayal and colleagues (Sayal *et al.*, 2009), the sample of 80 schools provide 85% power at the (2-sided) 5% level of significance to detect a difference between the trial arms of 0.3 standard deviations (effect size) on the teacher-reported SDQ-Total Difficulties score [equivalent to a difference of just under 2 points on the raw scale based on standard deviation of 5.9 in the British norming sample (Goodman, 2015)].

All analyses, performed using Stata software (StataCorp, 2015), were pre-specified in a statistical analysis plan that was reviewed by the independent DMC and TSC.

Baseline characteristics of the schools, teachers, and children were summarised for each trial arm. The characteristics of participating schools were compared with the school census for England 2012 (Department of Education). The trial outcomes at follow-up were compared using the intention-to-treat principle; children were analysed strictly according to the trial arm to which their school was randomised. The main findings presented are based on analyses of complete cases. In addition, we carried out sensitivity analyses based on 50 multiply imputed datasets using the chained equations approach (Raghunathan *et al.*, 2001).

Quantitative outcomes were compared between trial arms using random effects linear regression models and binary outcomes were compared using marginal logistic regression models using Generalised Estimating Equations with information sandwich (‘robust’) estimates of standard error assuming an exchangeable correlation structure. These methods allow for the correlation of children's outcome scores within schools. The primary analyses were those in which potential confounders were adjusted for, specified a priori in the analysis plan as follows: cohort, the three school/class level factors used to balance the randomisation, child gender, baseline score of the outcome, index of multiple deprivation score based on child's address, number of children living in their household and whether the child's household was rented. The latter three of these nine prognostic factors had a large amount of missing data because they were parent-reported. Adjusting for these would have resulted in the loss of a quarter of the sample in the complete case analyses. On this basis, and after discussion with our DMC (5 June 2017), we agreed on the primary analysis as the complete case analysis adjusted for only the six a-priori prognostic factors that were teacher-reported (CC1). For completeness, we also report the findings from the fully adjusted complete case analysis (CC2) and the fully adjusted analysis of imputed data with all nine prognostic factors included (MI).

For all outcomes, tests of interaction were used to assess whether there was evidence that the effect of the TCM intervention differed across the three follow-up timepoints. Where the interaction effect is statistically significant at the 5% level we report the effect at each timepoint, otherwise, we report an overall estimate of the intervention effect across the full 30-month follow-up period. We used this approach to avoid running an unnecessarily large number of statistical tests for these outcomes. For some outcomes, there was little evidence that the size of the true effect differed across the three follow-up time points so we reported a pooled overall estimate for those. This is an appropriately concise approach for reporting the findings.

In an ancillary analysis, we used the two-stage least squares instrumental variable method (Dunn and Bentall, 2007) to calculate the complier average causal effect (CACE) estimate of the intervention effect on the primary outcome teacher-reported SDQ-Total Difficulties score that would have occurred if all the teachers in the intervention arm had attended all six TCM training sessions.

Tests of interaction were also used in a priori pre-specified exploratory analyses to assess whether the effect of TCM on the primary outcome differs across sub-groups defined by the following potential moderator variables: school or child level deprivation status (in bottom 2 deciles *v.* otherwise), whether the child scored in the struggling range on the teacher-reported SDQ-Total Difficulties score at baseline, length of study teacher's experience (more than 5 years *v.* 5 years or less), Key Stage status (Key Stage 1 *v.* Key Stage 2), child's gender, and cohort status.

For the economic analysis, costs and cost-effectiveness of trial arms at final follow-up were compared. Total costs for each individual were calculated by applying nationally applicable unit costs to each item of service use reported, with costs incurred after 12 months discounted at the NICE-recommended rate of 3.5% annually (National Institute for Health and Clinical Excellence, 2008). Detailed information on the availability of economic data, unit costs applied, reported service use, and economic analyses is provided in the online Supplementary material. Cost-effectiveness was explored in terms of the SDQ and assessed by calculating incremental cost-effectiveness ratios (ICER; the additional cost of one intervention compared with another divided by the additional effect). The uncertainty of these estimates was explored by constructing cost-effectiveness acceptability curves (CEAC) (Fenwick *et al.*, 2001) to show the probability of TCM being cost-effective for a range of willingness to pay thresholds.

## Results

We recruited a total of 80 schools; 40 were allocated to each arm across all three cohorts (10 and 5 schools in the TCM and TAU arms, respectively, for Cohort 1; 15 and 15 for Cohort 2; and 15 and 20 for Cohort 3). During the trial, some schools did not provide teacher-completed data at the 9-month (*n* = 1), 18-month (*n* = 2), and 30-month (*n* = 1) assessments. In addition, one intervention school withdrew from the trial after completing the 18-month assessment (Fig. 1). A total of 2075 children were recruited to the trial (1037 in the intervention arm and 1038 in the control arm). A further 113 were either opted out by their parents (107) or ineligible (6). We lost contact with 271 (13%) children over the 30-month follow-up period and two parents withdrew permission for parent-reported outcomes but permitted collection of teacher- and child-reported outcomes.

Compared with the national average (Department of Education, 2012) participating schools had similar class sizes (mean 27.4 *v.* 26.8) and eligibility for free school meals (18.3% *v.* 19.3%), but included fewer voluntary controlled schools (5% *v.* 14.4%), and more community (61.3% *v.* 55.3%) and academy schools (10% *v.* 6%). Baseline characteristics for schools, teachers, and pupils were generally balanced between the two arms (Table 1), which was maintained at follow-up. Teachers in the intervention arm were less experienced (50% with more than five years’ experience *v.*. 67.5%). As Table 1 shows, the control arm contained relatively few children in Reception (8.5%) while over a third of children in the intervention arm were in Year 3 (37.%). Primary outcome data were collected at 9-, 18-, and 30-months follow-up for 96%, 89%, and 85% of participants, respectively. The proportion of children scoring in the struggling range on the teacher-reported SDQ-Total Difficulties score in both arms approached the expected 20% (cut point at the 80th centile) but was lower according to parent-report (16.5% TCM, 15.5% TAU), which suggests we lacked parental data on some vulnerable children. No serious adverse events were reported in either trial arm.

**Table 1.** Baseline characteristics by trial arm status

Variable	Intervention (TCM)	Control (TAU)
	*N*_S_ = 40	*N*_S_ = 40
School (cluster) characteristics		
Rural *v.* urban school, *n* (%)		
Rural, *n* (%)	18 (45.0)	19 (47.5)
Urban, *n* (%)	22 (55.0)	21 (52.5)
Education Key Stage^a^		
Key stage 1, *n* (%)	20 (50.0)	21 (52.5)
Key stage 2, *n* (%)	20 (50.0)	19 (47.5)
% eligible for free school meals, median (IQR)	12 (8–24)	14 (10–23)
Index of multiple deprivation score, median (IQR)	0.17 (0.08–0.24)	0.16 (0.10–0.27)
Teacher (cluster) characteristics	*N*_T_ = 40	*N*_T_ = 40
More than 5 years of teaching, *n* (%)	20 (50.0)	27 (67.5)
Age in years, mean (s.d.)	34.5 (9.0)	31.4 (8.7)
Female, *n* (%)	32 (80.0)	33 (82.5)
Newly qualified, *n* (%)	2 (5)	0 (0)
Has management position, *n* (%)	4 (10)	2 (5)
Teacher Self-efficacy Questionnaire		
Student Engagement subscale, mean (s.d.)	6.8 (1.0)	7.1 (1.0)
Instructional Practice subscale, mean (s.d.)	6.9 (1.0)	7.2 (0.9)
Classroom Management subscale, mean (s.d.)	7.3 (0.9)	7.5 (0.9)
Maslach Burn-Out Inventory		
Exhaustion, mean (s.d.)	2.9 (1.4)	2.5 (1.4)
Cynicism, mean (s.d.)	1.2 (1.0)	1.1 (1.0)
Professional Efficacy, mean (s.d.)	4.2 (1.0)	4.6 (0.8)
Everyday Feelings Questionnaire (teacher well-being), mean (s.d.)	17.2 (6.9)	13.9 (6.6)
Pupil characteristics		
	*N*_P_ = 1037	*N*_P_ = 1038
Female, *n* (%)	483 (46.6)	491 (47.3)
Age in years at last birthday, mean (s.d.; range)	6.2 (1.4; 4–9)	6.4 (1.3; 4–8)
Year group		
Reception	182 (17.6)	88 (8.5)
Year 1	176 (17.0)	192 (18.5)
Year 2	135 (13.0)	275 (26.5)
Year 3	389 (37.5)	220 (21.2)
Year 4	155 (14.9)	263 (25.3)
Ethnicity		
	*N*_P_ = 721	*N*_P_ = 701
White, *n* (%)	689 (95.6)	663 (94.6)
Black, *n* (%)	4 (0.6)	4 (0.6)
Asian, *n* (%)	5 (0.7)	11 (1.6)
Mixed, *n* (%)	20 (2.8)	18 (2.6)
Other, *n* (%)	3 (0.4)	5 (0.7)
	*N*_P_ = 595	*N*_P_ = 502
Eligible for free school meals, *n* (%)	70 (11.8)	64 (12.7)
	*N*_P_ = 860	*N*_P_ = 844
Index of multiple deprivation score, median (IQR)	0.16 (0.08–0.64)	0.15 (0.09–0.25)
	*N*_P_ = 770	*N*_P_ = 747
Number of children in household		
1, *n* (%)	125 (16.2)	122 (16.3)
2, *n* (%)	403 (52.3)	389 (52.1)
3, *n* (%)	175 (22.7)	158 (21.2)
4, *n* (%)	45 (5.8)	49 (6.6)
5 or more, *n* (%)	22 (2.9)	29 (3.9)
	*N*_P_ = 766	*N*_P_ = 744
Lives in rented housing, *n* (%)	475 (62.0)	423 (56.9)
	*N*_P_ = 758	*N*_P_ = 734
Parent's highest qualification		
None, *n* (%)	29 (3.8)	46 (6.3)
GSCE or equivalent/A Level or equivalent, *n* (%)	377 (49.7)	377 (51.4)
University Degree or equivalent and above, *n* (%)	352 (46.4)	311 (42.4)
	*N*_P_ = 1036	*N*_P_ = 1038
SDQ Total Difficulties score (teacher report), mean (s.d.) Raised if 12 +	6.8 (5.6)	6.6 (6.1)
SDQ Total Difficulties score in struggling range^b^ (teacher report), *n* (%)	206 (19.9)	200 (19.3)
SDQ Behaviour score (teacher report), mean (s.d.) Raised if 3 +	0.8 (1.5)	0.9 (1.6)
SDQ Emotions score (teacher report), mean (s.d.) Raised if 3 +	1.5 (2.0)	1.4 (2.1)
SDQ Overactivity score (teacher report), mean (s.d.) Raised if 6 +	3.3 (3.0)	3.1 (3.2)
SDQ Peer Relationships score (teacher report), mean (s.d.) Raised if 3 +	1.2 (1.5)	1.1 (1.7)
SDQ Pro-social score (teacher report), mean (s.d.) Low if <6	7.3 (2.5)	7.6 (2.4)
SDQ Impact score > 0 (teacher report), *n* (%) Raised if 1 +	395 (38.1)	373 (35.9)
	*N*_P_ = 733–752	*N*_P_ = 706–715
SDQ Total Difficulties score (parent report), mean (s.d.) Raised if 14 +	7.8 (6.0)	7.8 (5.9)
SDQ Total Difficulties score in struggling range^c^ (parent report), *n* (%)	124 (16.5)	111 (15.5)
SDQ Behaviour score (parent report), mean (s.d.) Raised if 3 +	1.5 (1.6)	1.4 (1.6)
SDQ Emotions score (parent report), mean (s.d.) Raised if 4 +	1.8 (2.0)	1.8 (2.0)
SDQ Overactivity score (parent report), mean (s.d.) Raised if 6 +	3.3 (2.6)	3.3 (2.6)
SDQ Peer Relationships score (parent report), mean (s.d.) Raised if 3 +	1.2 (1.6)	1.3 (1.6)
SDQ Pro-social score (parent report), mean (s.d.) Low if <6	8.4 (1.7)	8.5 (1.7)
SDQ Impact score > 0 (parent report), *n* (%) Raised if 1 +	244 (33.3)	209 (29.6)
	*N*_P_ = 1036	*N*_P_ = 1038
Pupil Behaviour Questionnaire, mean (s.d.)	2.0 (2.4)	1.9 (2.4)
	*N*_P_ = 1025	*N*_P_ = 1028
How I Feel About My School, mean (s.d.)	10.9 (2.5)	11.1 (2.3)
	*N*_P_ = 746	*N*_P_ = 713
Assessment of Pupil Reading Level (parent report)		
Fluent Reader, *n* (%)	320 (42.9)	349 (48.9)
	*N*_P_ = 753	*N*_P_ = 713
Assessment of Pupil's Relationship with Teacher (parent report)		
Good Relationship, *n* (%)	632 (83.9)	622 (87.2)
	*N*_P_ = 731	*N*_P_ = 703
Assessment of Parent's Relationship with Teacher (parent report)		
Good Relationship, *n* (%)	465 (63.6)	485 (69.0)

a Education Key Stage 1 covers Reception to Year 2 for children aged 4–7; Key Stage 2 covers Years 3–6 for children aged 7–8.

b Struggling defined as scoring 12 or more out of 40.

c Struggling defined as scoring 14 or more out of 40.

SDQ norms from www.sdqinfo.com.

*N*_S_ – number of schools in denominator.

*N*_T_ – number of teachers in denominator.

*N*_P_ – number of pupils in denominator.

Table 2 summarises the comparison between the trial arms at follow-up for the primary outcome measure. TCM improved child mental health according to the teacher-reported SDQ-Total Difficulties score by 1.0 point (95% CI 0.1–1.9; *p* = 0.03) at the 9-month follow-up. There was no evidence, however, of an effect at the 18-month (*p* = 0.85) and 30-month follow-ups (*p* = 0.23). The findings from the fully-adjusted complete case sensitivity analysis were similar except there was only weak evidence of an effect at 9-months on the teacher reported SDQ-Total Difficulties. Post hoc analysis showed that this is because a large number of children lost from the fully-adjusted analysis due to missing data on the three parent-reported potential confounders were also those in whom the TCM effect was greatest. The intervention effect on teacher-reported SDQ-Total Difficulties was −1.6 (95% CI −2.8 to −0.4) for the 534 children with missing data on the three parent-reported potential confounders and −0.8 (95% CI −1.7 to 0.1) for the remaining 1467 children with complete data. Finally, the fully adjusted analysis of imputed data provided very similar results to our primary partially-adjusted analysis. All the remaining findings are based on the approach used in the partially-adjusted analysis.

**Table 2.** Main comparison on teacher-reported SDQ total difficulties score (primary outcome) using different approaches for handling missing data

Follow-up	Intervention	Control	CC1 – primary analysis					CC2 – sensitivity analysis				MI –sensitivity analysis			
	Mean (s.d.)	Mean (s.d.)	Adjusted mean diff. (I – C)					Adjusted mean diff. (I – C)				Adjusted mean diff. (I – C)			
			*N*	Est.	95% CI	*p*	ICC	*N*	Est.	95% CI	*p*	*N*	Est.	95% CI	*p*
9-months	5.5 (5.4)	6.2 (6.2)	2001	−1.0	−1.9 to −0.1	0.03	0.180	1467	−0.8	−1.6 to 0.1	0.09	2075	−1.0	−1.9 to −0.2	0.02
18-months	6.7 (6.9)	6.5 (6.3)	1848	−0.1	−1.5 to 1.2	0.85	0.179	1371	−0.2	−1.5 to 1.1	0.75	2075	−0.1	−1.4 to 1.1	0.82
30-months	6.1 (6.0)	6.5 (6.6)	1756	−0.7	−1.9 to 0.4	0.23	0.121	1318	−0.6	−1.8 to 0.5	0.30	2075	−0.8	−1.9 to 0.3	0.14

CC1 – partially-adjusted complete case analysis (primary analysis); CC2 – fully-adjusted complete case analysis; MI – fully-adjusted analysis of imputed data.

ICC – Intra-cluster (intra-school) correlation coefficient from crude (unadjusted) analysis.

Thirty-six (90%) of the 40 teachers in the intervention arm attended four or more TCM sessions; 23 attended all six. Findings from the complier average causal effect analysis were almost identical to the primary intention-to-treat analysis, which suggests that the estimated effects would have been no different had all the teachers in the TCM arm attended all six sessions.

Tests of interaction indicated that TCM led to greater reductions in the teacher-reported SDQ-Total Difficulties score at 9 months (interaction *p* < 0.001) for children who were classified by their teacher as struggling with their mental health at baseline (mean difference = −2.6; 95% CI −4.6 to −0.6) than for children who were not (mean difference = −0.4; 95% −1.2 to 0.4). A subgroup effect was also found at 30 months (*p* < 0.001) but not 18 months (*p* = 0.10). TCM may also have greater benefits at 30-months (interaction test *p* = 0.02) for children taught by teachers with more than 5 years’ experience (mean difference on teacher-reported SDQ-Total Difficulties score = −2.1; 95% CI −3.8 to −0.4) compared with those with 5 years or less experience (mean difference = 0.3; 95% CI −1.3 to 1.9). TCM appeared more effective for Cohort 2 schools than those in Cohorts 1 and 3 (interaction *p* = 0.02) but there was little evidence of sub-group effects for the other potential moderator variables.

Table 3 summarises the findings from the teacher-reported secondary outcomes. There was evidence, based on the PBQ score, of reduced disruptive behaviour across all three follow-ups (*p* = 0.04). Likewise, there was evidence that TCM reduces the percentage of children that are classified as struggling according to the SDQ-Total Difficulties score (*p* = 0.05) and reduces the Inattention/Overactivity score (*p* = 0.02) across all waves. At 9 months only there was also evidence of a reduction in peer relationship problems (*p* = 0.02) and an improvement in pro-social behaviour (*p* = 0.02). Finally, there was little evidence of effects on teacher-reported Emotions and Impact, parents’ assessment of their child's mental health or the child-reported outcome *HIFAMS* (Table 4).

**Table 3.** Comparison of teacher-reported secondary outcomes

Outcome	Intervention arm (I)	Control arm (C)	Adjusted mean difference/odds ratio^a^			
	Mean (s.d.)/%	Mean (s.d.)/%	Estimate	95% CI	*p* value	ICC
SDQ Total difficulties score in struggling range^b^						
9–30-months	16.7%	19.2%	0.70	0.48–0.99	0.05	0.062
SDQ Behaviour score						
9-months	0.7 (1.5)	0.9 (1.6)	−0.1	−0.3 to 0.1	0.27	0.092
18-months	1.0 (1.8)	0.9 (1.7)	−0.03	−0.3 to 0.3	0.86	0.117
30-months	0.9 (1.5)	1.0 (1.8)	−0.2	−0.5 to 0.1	0.18	0.104
SDQ Emotions score						
9-months	1.3 (1.9)	1.5 (2.2)	−0.3	−0.6 to 0.1	0.14	0.202
18-months	1.7 (2.2)	1.6 (2.1)	0.1	−0.3 to 0.6	0.63	0.179
30-months	1.6 (2.1)	1.6 (2.1)	−0.005	−0.4 to 0.3	0.98	0.090
SDQ Overactivity score						
9–30-months	2.7 (2.9)	2.8 (3.0)	−0.4	−0.7 to −0.1	0.02	0.079
SDQ Peer relationships score						
9-months	0.8 (1.4)	1.0 (1.7)	−0.2	−0.4 to −0.03	0.02	0.119
18-months	1.1 (1.7)	1.0 (1.6)	0.1	−0.2 to 0.4	0.62	0.131
30-months	1.1 (1.6)	1.1 (1.7)	−0.07	−0.4 to 0.2	0.60	0.098
SDQ Pro-social score						
9-months	8.2 (2.3)	8.0 (2.3)	0.4	0.1– 0.8	0.02	0.251
18-months	7.8 (2.4)	8.0 (2.3)	−0.1	−0.6 to 0.4	0.67	0.204
30-months	8.1 (2.2)	7.6 (2.3)	0.5	−0.03 to 1.0	0.06	0.164
SDQ Impact score >0						
9–30-months	34.5%	37.3%	0.80	0.61–1.05	0.11	0.048
Pupil behaviour questionnaire						
9–30-months	1.8 (2.4)	1.9 (2.6)	−0.3	−0.5 to −0.01	0.04	0.042

a Mean difference reported for quantitative outcomes and odds ratios reported for binary outcomes.

b Struggling scoring 12 or more out of 40.

The sample size for 9-month assessments is 981 in the intervention arm and 1020 in the control arm.

The sample size for 18-month assessment is 894 in the intervention arm and 954 in the control arm.

The sample size for 30-month assessment is 856 in the intervention arm and 900 in the control arm.

ICC – Intra-cluster (intra-school) correlation coefficient from crude (unadjusted) analysis.

**Table 4.** Comparison of parent- and child-reported secondary outcomes

Outcome	Intervention arm (I)	Control arm (C)	Adjusted mean difference/odds ratio^a^			
	Mean (s.d.)/%	Mean (s.d.)/%	Estimate	95% CI	*p* value	ICC
Parent-reported outcomes						
SDQ Total difficulties score						
9–30-months	7.7 (6.5)	7.6 (6.4)	0.1	−0.3 to 0.5	0.64	0.008
SDQ Total difficulties score in struggling range^b^						
9–30-months	17.5%	15.4%	1.24	0.92–1.67	0.16	0.030
SDQ Behaviour score						
9–30-months	1.4 (1.7)	1.3 (1.6)	0.03	−0.1 to 0.1	0.63	0.025
SDQ Emotions score						
9–30-months	2.1 (2.3)	2.0 (2.3)	0.02	−0.2 to 0.2	0.86	0.017
SDQ Overactivity score						
9–30-months	3.0 (2.7)	3.0 (2.6)	0.1	−0.1 to 0.3	0.30	0.004
SDQ Peer relationships score						
9–30-months	1.3 (1.7)	1.3 (1.8)	−0.03	−0.2 to 0.1	0.66	0.034
SDQ Pro-social score						
9–30-months	8.6 (1.7)	8.7 (1.7)	−0.04	−0.2 to 0.1	0.54	0.002
SDQ Impact score >0						
9–30-months	33.3%	30.8%	0.94	0.71–1.25	0.69	0.035
Assessment of pupil reading level						
Fluent reader						
9–30-months	64.3%	67.5%	0.96	0.75–1.23	0.75	0.081
Assessment of pupil's relationship with teacher						
Good relationship						
9–30-months	85.6%	84.4%	1.20	0.93–1.54	0.16	0.028
Assessment of parent's relationship with teacher						
Good relationship						
9– 30-months	73.1%	73.3%	1.12	0.87–1.45	0.39	0.039
Child-reported outcomes						
How I feel about my school						
9-months	10.8 (2.5)	10.9 (2.4)	0.02	−0.3 to 0.3	0.89	0.077
18-months	10.5 (2.5)	10.4 (2.8)	0.3	−0.1 to 0.7	0.17	0.106
30-months	10.4 (2.8)	10.3 (2.8)	0.2	−0.2 to 0.7	0.35	0.111

a Mean difference reported for quantitative outcomes and odds ratios reported for binary outcomes.

b Struggling scoring 14 or more out of 40.

The sample size for 9-month parent-reported assessments ranges from 624 to 646 in the intervention arm and 637–642 in the control arm.

The sample size for 18-month parent-reported assessments ranges from 600 to 611 in the intervention arm and 606–617 in the control arm.

The sample size for 30-month parent-reported assessments ranges from 550 to 558 in the intervention arm and 557–569 in the control arm.

The sample size for 9-month child-reported assessment is 991 in the intervention arm and 995 in the control arm.

The sample size for 18-month child-reported assessment is 943 in the intervention arm and 943 in the control arm.

The sample size for 30-month child-reported assessment is 864 in the intervention arm and 896 in the control arm.

ICC – Intra-cluster (intra-school) correlation coefficient.

Table 5 summarises total costs and outcomes at final follow-up for those children with full economic data (service use and outcome data), and reports the results of both unadjusted and partially adjusted complete case analyses (the primary analysis, CC1). Observed mean total costs of services used over the 30-month follow-up were slightly lower for the intervention arm (£524.16) compared with the control arm (£528.14). However, this difference was not statistically significant (adjusted mean difference: £30.24, 95% CI −£140.98 to £201.47, *p* value = 0.73). Observed mean SDQ-Total Difficulties scores for the sample of children with full economic data, were slightly lower (better outcomes) in the intervention (5.17) than the control (5.39) arms but these differences were also not statistically significant (adjusted mean difference −0.54, 95% CI −1.68 to 0.61, *p* value = 0.36).

**Table 5.** Mean costs (£) and outcome per participant over the 30-months follow-up period (CC1)*

	Intervention arm (I) (*N* = 507)	Control arm (C) (*N* = 500)	Unadjusted mean difference (I-C)	Adjusted mean difference (I-C)*		
	Mean (s.e.)	Mean (s.e.)		Estimate	95% CI	*p* value
Costs (£)						
Baseline	119.82 (15.20)	115.99 (10.87)	3.83	−11.67	−49.90 to 26.55	0.55
Intervention	11.52 (0.00)	0.00 (0.00)	11.52	.	.	.
Hospital	373.49 (0.70)	354.59 (36.80)	18.90	21.60	−70.13 to 113.33	0.64
Community	60.24 (0.70)	74.91 (8.26)	−14.67	−12.42	−24.12 to 9.28	0.26
Medication	2.93 (0.70)	19.08 (7.06)	−16.16	−9.11	−25.93 to 7.71	0.29
Accommodation	0.00 (0.00)	49.36 (27.66)	−49.63	−49.70	−127.21 to 27.80	0.21
Productivity loss	76.60 (57.18)	27.67 (40.58)	48.93	−70.41	−42.51 to 183.33	0.22
Total	524.16 (90.60)	528.14 (64.31)	−3.98	30.24	−140.98 to 201.47	0.73
Outcome						
SDQ total difficulties	5.17 (5.49)	5.39 (6.05)	−0.22	−0.54	−1.68 to 0.61	0.36

*CC1 – partially adjusted complete case analysis (primary analysis).

**Fig. 2.**
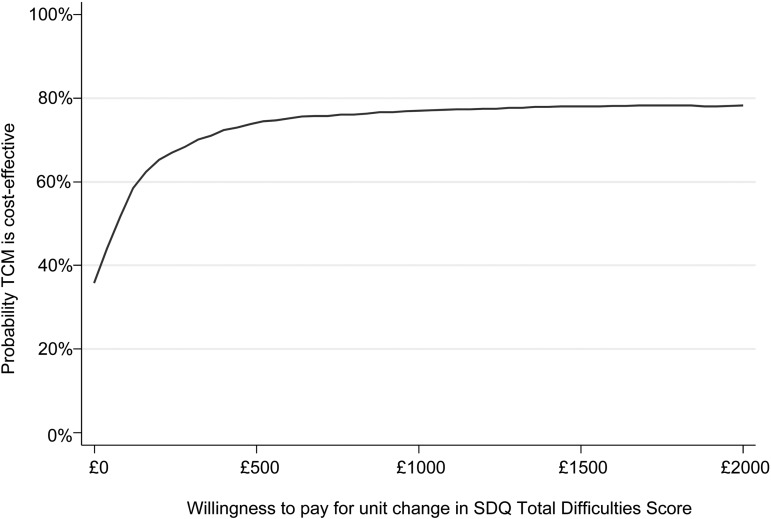
Cost-effectiveness acceptability curve showing the probability that TCM is cost-effective compared to TAU for different values of willingness to pay thresholds (CC1)*. *CC1 – partially adjusted complete case analysis (primary analysis).

The observed lower costs and better outcomes in the intervention group generate an ICER of -£29.70 per unit improvement in SDQ, which suggests that the intervention dominates TAU and is cost-effective. The CEAC for the partially-adjusted complete case analysis (CC1) suggests that the probability of the intervention being cost-effective compared with TAU ranges from just under 40% at a zero willingness to pay for a unit improvement in SDQ-Total Difficulties score, to nearly 80% at a £5000 willingness to pay threshold (Fig. 2) and is 50% or higher at values of £70 and above. Sensitivity analyses using fully-adjusted (CC2) and imputed (MI) datasets were similar (see online Supplementary material).

## Discussion

We detected a small but statistically significant improvement in teacher-reported children's mental health at 9-months (primary outcome). Similar effect sizes have been reported on the same measure in before-and-after studies of attendance at the child and adolescent mental health services (Fugard *et al.*, 2015) and small effects from universal interventions are common and expected (Greenberg and Abenavoli, 2017). Almost all of the 95% confidence interval for the mean difference, however, lies below the initially assumed minimum clinically important difference (effect size of 0.3 or raw difference of 1.8), although it remains debatable whether public health trials should expect clinically important differences to be obtained on an initially healthy population. Furthermore, the children who appear to derive the greatest benefit were more likely to be missing data from parents, which may have reduced our ability to detect any effect of the intervention on parent SDQ.

The population influence of universal interventions may be differentiated across subgroups, with the same intervention acting to promote health for some, while preventing deterioration or actively treating others. Small population effects, therefore, do not necessarily demonstrate lack of effectiveness (Greenberg and Abenavoli, 2017). Indeed, the a priori planned exploratory subgroup analyses suggest that children with poorer mental health derived the most benefit according to teacher report. A recent multi-level meta-analysis of three TCM trials (Nye, 2017) also suggests a clinically and statistically significant effect on children with worse behaviour, a finding echoed in the recently published trial from North Carolina (Murray *et al.*, 2018), which was not included in the quoted meta-analysis. A similar pattern of differential response according to baseline mental health was also reported in an individual-level meta-analysis of trials of the parallel Incredible Years^®^ parent training intervention (Leijten *et al.*, 2018).

There is some evidence to suggest that TCM has a higher probability of being cost-effective compared with TAU over a wide range of values of willingness to pay for improvements in SDQ-Total Difficulties scores (£100–£1500). As is common with universal interventions, differences in both costs and effects in favour of TCM were small and it is not possible to draw a firm conclusion without knowing society's willingness to pay for improvements in SDQ-Total Difficulties score. At the start of the trial, there was no valid health-related quality of life measure for children under 8 years of age, but future economic modelling would enable the SDQ-Total Difficulties to be mapped onto the adult quality of life.

The effect of TCM on the primary outcome was not maintained at 18 and 30 months, which could mean that TCM has no long-term impact, or be due to the children's reaction to the teaching style of their subsequent teachers who had not accessed the course. The North Carolina trial reported that the positive change in school climate was also not sustained into the following year (Murray *et al.*, 2018). As the intra-cluster correlation coefficients were markedly larger for teacher-reported SDQ-Total Difficulties score (0.12–0.18) than the corresponding parent-reported score (0.06), variability in how teachers score their pupils may contribute additional noise at the two later follow-ups, which were scored by different teachers.

Children in the intervention arm were exposed to TCM strategies for a relatively short duration; teachers in our parallel process evaluation anticipated larger effects in subsequent years when they could apply the skills gained from TCM training during their planning and from the outset of the academic year (Ford *et al.*, Under review). The impact of the intervention might arguably increase in the year *after* the teacher attended the course, which we were unable to asses as our trial design followed the children rather than the teacher. As most effective universal programmes employ a whole school approach (Greenberg and Abenavoli, 2017), training all school staff to use the same strategies might amplify and sustain any initial impact on children's mental health that training a single teacher might have, as children could then benefit from the intervention throughout their school years. Both questions should be addressed empirically by training more than one member of staff per school and by following the children subsequently taught by these staff in the year after they have accessed the course. The North Carolina trial randomised 4–14 teachers per school from Kindergarten, First and Second Grade (4–7 years) to TCM immediately (*n* = 45) or the following year (*n* = 46). The study involved 1192 pupils, and selected across 3-year groups, but may have introduced contamination between arms since every school had at least one class allocated to the intervention (Murray *et al.*, 2018)

The small but sustained effects on disruptive behaviour (PBQ) and inattention/hyperactivity (SDQ) across all three follow-ups are interesting and warrant replication. The PBQ was used because it examines low-level disruption in the classroom, whilst the SDQ behaviour scale taps a broader range of more severe antisocial behaviours (fighting, disobedience, tantrums, lying and stealing), which may explain the lack of change in the latter. More than 40% of secondary school children reported in a survey that their classroom was too noisy to work in (Wilson *et al.*, 2007), while overactivity/inattentive traits predict poorer academic attainment at GCSE (Stergiakouli *et al.*, 2017). Children in poorly managed classrooms observe that disruptive behaviour commands staff attention, which may amplify later disruptive behaviour and disengagement from school with its attendant risks to health and education. Teacher training and professional development might usefully explore how classroom management style may influence children's mental health as well as their behaviour and attainment.

We failed to detect any influence of TCM on the HIFAMS measure. Some would recommend 8-years of age as the minimum (Schwab-Stone *et al.*, 1996) for reliable reporting on standardised measures of mental health, although increasingly researchers are seeking reports from younger children. The HIFAMS was developed for STARS and has demonstrated validity and moderate reliability among children as young as four when tested in this and two other samples (Allen *et al.*, 2017).

The validity of STARS derives from high retention of schools, teachers and pupils over 30 months, the delivery of TCM with fidelity by experienced practitioners trained and supervised by the programme developer, independent randomisation, and the use of a strongly validated and widely used primary outcome measure. High levels of attendance suggest that teachers valued TCM, while the participating schools were generalisable in terms of class size and eligibility for free school meals.

It was, however, impossible to mask teachers, risking response bias, particularly for the 9-month follow-up where intervention teachers were the primary respondents. Classroom observations in this and other trials of TCM suggest that attendance at the course is associated with changes in teachers behaviour as well as improved child compliance (Hutchings *et al.*, 2013; Hickey *et al.*, 2017; Murray *et al.*, 2018; Hayes *et al.*, Under review). Similarly, a recent meta-analysis that examined the effect of the Incredible Years^®^ Parenting programme found that effects were as strong when based on independent observations compared with parent-report (Menting *et al.*, 2013). In addition, outcomes at 18 and 30 months were completed by different teachers who did not attend the training and the decrease in both the teacher-reported SDQ Hyperactivity subscale and the PBQ across all follow-ups undermines the argument that the primary outcome findings can be wholly explained by reporting bias. Similarly, teachers working with the study children in the last 2 years of the trial may have been aware that a colleague had accessed the course in the first (intervention) or subsequent (control) year of the study, but this may not have equated to the knowledge of allocation. Interviews with Special Educational Needs Coordinators in some participating schools as part of our process evaluation revealed that few were aware of the school's involvement in the study and understanding of the study design was poor (Nye, 2017). Special Educational Needs Coordinators have a particular role in relation to behaviour management so might arguably be expected to be more aware of the study than their classroom-based colleagues. Furthermore, the wait-list design to access the course meant that a teacher accessed the course in all schools, so we can be reasonably confident that these teachers’ responses would face similar influences in both arms, even though we were not able to actively maintain masking of allocation.

Headteachers are used to considerable autonomy, and it was clear from our feasibility work that any attempt to control the selection of teachers would be a major disincentive to their school's participation in the study. There are two potential biases that might occur from headteachers selection of teachers to attend the course. If teachers were selected because they struggled with behaviour management, we might overestimate the impact of the intervention, while if selected because of a particular interested in socio-emotional well-being, we might underestimate the impact if interest correlates with skills, or overestimate the impact if interest correlates with receptiveness. As the selection of teachers preceded randomisation, it should not have compromised the internal validity of the study and reasonable balance was obtained on teacher characteristics (see Table 1). Our process evaluation involved interviews with headteachers and suggested that a number of reasons for their choice of the teacher to nominate, which included newly qualified teacher status, allocation of a class known to be particularly challenging or known interest in behaviour management (Ford *et al.*, Under review). In addition, if TCM were disseminated, the logistics of ensuring adequate teacher cover in schools means that headteachers would have to select who to send for training as it would be impossible to train whole schools simultaneously. Our method, therefore, presaged the likely process of any subsequent implementation, and so constitutes a fair test of the intervention.

Balance at the school level was excellent, but teachers in the control arm were more experienced and despite balance between the Key Stages of education, there was some imbalance in individual year groups. These differences were small, and we believe unlikely to influence the findings unduly.

Although we successfully obtained data from more than 70% of the parents at baseline, attrition was marked at follow up, and differential between those living in deprivation and those with poor mental health at baseline compared with their more affluent and healthier peers, which might have reduced the chance to detect any effect that TCM may have had at home. Differential loss to follow up in these overlapping populations is well-documented in child mental health research and does not necessarily influence findings (Wolke *et al.*, 2009). Response rates for the CA-SUS were particularly affected by low parent response at follow up. Whilst multiple imputations of the missing data did not markedly affect the results, any conclusions reached must be considered alongside this low response. Children respond differently to different situations and TCM targets the classroom rather than the home. Low levels of agreement between parents and teachers is common in child mental health research (Collishaw *et al.*, 2009) so that the lack of effect on parent-reported outcomes does not necessarily indicate lack of effectiveness in the school context.

In conclusion, our findings provide tentative evidence that TCM may be an effective and cost-effective child mental health intervention in the short term, particularly for primary school children who teachers identify as struggling. Future research should explore TCM as a whole school approach.

## Supplementary material

For supplementary material accompanying this paper visit https://doi.org/10.1017/S0033291718001484. click here to view supplementary material
