# Supplementary material for: The effectiveness and cost-effectiveness of the Incredible Years® Teacher Classroom Management programme in primary school children: results of the STARS cluster randomised controlled trial
Source: Psychol Med. 2018 Jul 18;49(5):828–42. doi: 10.1017/S0033291718001484 (PMC6425365; doi:10.1017/S0033291718001484)
Supplement: Supplementary file 1 [file S0033291718001484sup001.docx]

# Appendix

## Economic Evaluation

### Methods

#### Perspective

The economic evaluation took a broad public-sector perspective, including hospital services (inpatient, outpatient, accident and emergency), community health services (primary health care and community mental health services), medication and Local Authority accommodation, as well as productivity losses of parents relating to their child.

#### Service use and total costs

A brief self-report version of the Child and Adolescent Service Use Schedule (CA-SUS) (Barett *et al.*, 2006, Byford *et al.*, 1999) was used to collect service use data from parents at baseline, 9-, 18- and 30-months follow-up and was tested prior to implementation on a sample of families to ensure face validity and adequate coverage. The CA-SUS specifically measured inpatient hospital stays, outpatient or day-patient hospital appointments, visits to accident and emergency, contacts in the community with Child and Adolescent Mental Health Services or a Family Support Worker, appointments with any health professional in general practice (GP, nurse or health visitor), use of certain medication (methylphenidate, dexamphetamine and atomexetine), time spent in Local Authority foster care, respite care or residential care, and time taken off work by parents due to child’s behavioural difficulties or other worries.

A second, full standard interview version of the CA-SUS was also used with a random sample of 48 parents at 9- and 18-months follow-up in order to validate and supplement the briefer self-complete version and will form part of a future publication, along with work exploring longer-term implications of TCM using decision analytical modelling (see Economic analyses section below).

Unit costs were for the 2014/15 financial year and costs falling after 12-months were discounted by 3.5%, as recommended by the National Institute for Health and Care Excellence (NICE) (National Insitute for Health and Clinical Excellence, 2013).

The intervention cost was calculated using a standard micro-costing (bottom-up) approach (Netten A., 1998), and was based on the costs of training the Incredible Years group leaders and costs of training and supervision of teachers receiving TCM. The allocation of intervention costs to individual pupils assumed average class size of 30 students, and teachers staying in the workforce for 5 years, as per published reports of trends in teaching (Barmby, 2006).

A nationally applicable unit cost was applied to all other items of service use reported to calculate the total cost for each participant for the duration of the trial. Costs for NHS hospital contacts were sourced from NHS reference costs 2014-15 ((DH), 2015). Costs contained in the annual unit costs of health and social care publication were applied to community-based health and local authority accommodation services (Curtis, 2015). The cost of medications was based on averages listed in the British National Formulary (Royal Pharmaceutical Society of Great Britain, 2016). Productivity losses were valued using workers’ median gross earnings as listed in the ONS Annual Survey of Hours and Earnings (Office for National Statistics, 2015). A summary of unit costs applied is listed in Table 1.

#### **Economic analyses**

Economic analyses explored three scenarios as per the analysis of clinical data. The primary analysis (CC1) was based on complete cases and partially adjusted for pre-specified potential confounders (cohort, child gender, and the three school/class level factors used to balance randomisation) and for baseline costs and outcomes, as appropriate. In addition, fully adjusted sensitivity analyses (same confounders as CC1 plus index of multiple deprivation score based on child’s address, number of children living in the household and whether the child’s household was rented) were conducted for complete cases (CC2) and 50 multiply imputed datasets (MI) using the chained equations approach.

Differences in service use between trial arms at 30-months follow-up were compared descriptively. Despite the often skewed nature of costs, analyses compared mean costs between groups using standard parametric tests and assessed the robustness of results using bias-corrected accelerated non-parametric bootstrapping. This approach allows inferences to be made about the arithmetic mean as opposed to logarithmic transformation or non-parametric tests and is more meaningful from a budgetary perspective.

Cost-effectiveness was explored in terms of the SDQ Total Difficulties score. The incremental cost effectiveness ratio (ICER) was calculated based on parameter estimates from bivariate random-effects linear regression models that model the costs and SDQ outcomes simultaneously, taking account of the hierarchical structure of the data in cluster randomised trials. The parameters from the bivariate model were used to construct the cost-effectiveness acceptability curve (CEAC) to show the probability of the intervention being cost-effective for a range of possible values of willingness to pay per unit improvement in the SDQ.

Data from the trial will be supplemented with data from the literature to extrapolate beyond the length of the trial using decision analytic modelling techniques to explore the longer-term cost and cost-effectiveness implications of TCM compared to TAU and to model potential cost savings in the longer term. Additionally, attempts will be made to model expected cost-utility (cost per QALY approach preferred by NICE)(National Insitute for Health and Clinical Excellence, 2013) by using existing datasets to map the results of the SDQ onto an appropriate utility scale. This work is ongoing and will be published separately.

### Results

CA-SUS data on service use for the entire follow-up were available for 507 (48.9%) participants in the intervention arm and 500 (48.2%) participants in the control arm, after removing influential outliers, i.e. cases with total costs in the 99^th^ percentile that make a significant difference to the results.

#### Service use

Use of services over the 30-month follow-up are reported in Table 2. The use of health services was broadly similar across the two arms. Accommodation services were used only by those in the control arm, but these were only used by less than 1% of the sample.

#### Primary analysis (CC1)

Observed mean costs were lower (adjusted mean difference: £30.24, 95% CI: -£140.98 to £201.47, p-value=0.73) while outcomes were better (adjusted mean difference: -0.54, 95% CI -1.68 to 0.61, p-value=0.36) in the intervention arm compared to controls. Figure 1, the scatterplot of bootstrapped mean differences in costs and outcome shows that majority of the scatter points indicate an improvement in the SDQ Total Difficulties score and lie in the northeast (50%; more effective, more costly) and southeast (29%; more effective, less costly) quadrants. The remaining scatter points show poorer outcomes and fall in the northwest (14%; less effective, more costly) and southwest (7%; less effective, less costly) quadrants. The CEAC indicates the probability of the intervention being cost-effective ranges from just under 40% at a zero willingness to pay for a unit improvement in SDQ total difficulties score, to nearly 80% at a £1500 willingness to pay threshold (Figure 2).

#### Sensitivity analyses (CC2, MI)

Two sensitivity analyses were carried out: CC2- fully adjusted complete case analysis; and MI- fully adjusted analysis of imputed data. Results from both are presented in Table 3.

There were no statistically significant between-group differences in costs or outcomes for either of the sensitivity analyses. Adjusted mean difference of total costs (£16.44, 95% CI -£155.32 to £188.21, p-value 0.85) and outcomes (-0.51, 95% CI -1.66 to 0.63, p-value=0.38) for CC2 were similar to that of the primary analysis, supporting the high probability of TCM being cost-effective for a range of willingness to pay thresholds per unit improvement in SDQ (Figure 2).

Imputation of missing data showed a larger difference in both costs (adjusted mean difference £252.00, 95% CI -£221.00 to £725.00, p-value=0.30) and outcomes (adjusted mean difference -0.57, 95% CI -1.58 to 0.46, p-value=0.28) and had a negative impact on the probability of TCM being cost-effective, but only at the lower levels of willingness to pay thresholds (Figure 2).

Table 1. Unit costs and sources used in economic evaluation

| Service | Unit cost or range (£) | Source | Notes |
| --- | --- | --- | --- |
| Inpatient hospital (per night) | 488.00 – 674.00 | [NHS reference costs](https://www.gov.uk/government/uploads/system/uploads/attachment_data/file/480791/2014-15_National_Schedules.xlsx)((DH), 2015) | Weighted average of short and long stay |
| Outpatient hospital (per appointment) | 180.00 | [NHS reference costs](https://www.gov.uk/government/uploads/system/uploads/attachment_data/file/480791/2014-15_National_Schedules.xlsx)((DH), 2015) |  |
| Accident & Emergency (per attendance) | 152.00 | [NHS reference costs](https://www.gov.uk/government/uploads/system/uploads/attachment_data/file/480791/2014-15_National_Schedules.xlsx)((DH), 2015) |  |
| Community mental health (per appointment) | 43.00 | [Curtis 2015](http://www.pssru.ac.uk/project-pages/unit-costs/2015/index.php?file=full)(Curtis, 2015) | Assuming 30-minute appointment with CAMHS/Family Support Worker |
| General practice (per appointment) | 18.91 | [Curtis 2015](http://www.pssru.ac.uk/project-pages/unit-costs/2015/index.php?file=full)(Curtis, 2015) | Average of GP and nurse; assuming 10-minute consultation |
| Medication (per mg) | 0.02 – 0.19 | [BNF 70](https://www.medicinescomplete.com/mc/bnf/current/)(Royal Pharmaceutical Society of Great Britain, 2016) | Assuming recommended national guidelines on dosage |
| Accommodation (per day) | 317.79 | [Curtis 2015](http://www.pssru.ac.uk/project-pages/unit-costs/2015/index.php?file=full)(Curtis, 2015) | Average of Local Authority foster care, private foster care and private residential care |
| Productivity loss (per day) | 74.00 | [ONS Annual Survey of Hours and Earnings](https://www.ons.gov.uk/employmentandlabourmarket/peopleinwork/earningsandworkinghours/bulletins/annualsurveyofhoursandearnings/2015provisionalresults#average-earnings)(Office for National Statistics, 2015) | Based on part-time workers’ median gross earnings |

Table 2. Service use over 30-months follow-up

| Service | Unit | Intervention arm (I)  (N=507) | | | Control arm (C)  (N=500) | | |
| --- | --- | --- | --- | --- | --- | --- | --- |
|  |  | mean (SD) | range | % using | mean (SD) | range | % using |
| Inpatient hospital | no. nights | 0.09 (0.56) | 0-7 | 5.1 | 0.14 (1.20) | 0-23 | 4.2 |
| Outpatient hospital | no. appointments | 1.31 (2.51) | 0-20 | 38.5 | 1.10 (2.27) | 0-17 | 35.0 |
| Accident & Emergency | no. visits | 0.49 (1.09) | 0-13 | 28.8 | 0.51 (1.05) | 0-10 | 29.6 |
| Community mental health | no. appointments | 0.38 (2.08) | 0-31 | 8.1 | 0.65 (5.01) | 0-104 | 9.6 |
| General practice ^a^ | no. appointments | 2.23 (2.85) | 0-30 | 66.9 | 2.38 (3.07) | 0-33 | 67.0 |
| Medication ^b^ | % using | . | . | 0.4 | . | . | 1.2 |
| Accommodation ^c^ | no. days | 0.00 (0.00) | 0-0 | 0.0 | 0.15 (2.63) | 0-56 | 0.4 |
| Productivity loss | no. days taken off by parent due to child’s behaviour or other worries | 1.01 (16.54) | 0-370 | 5.52 | 0.36 (2.84) | 0-49 | 5.0 |

^a^ Includes appointments with any practitioner in the general practice (e.g. GP, practice nurse, health visitor)

^b^ Includes use of methylphenidate, dexamfetamine and atomexetine

^c^ Includes time spent in Local Authority foster care, respite care and residential care

Table 3. Results of sensitivity analyses

|  | **Intervention arm (I)** | **Control arm (C)** | **CC1 - primary analysis**  **Adjusted mean difference (I-C)**  **(N=1007)** | | | **CC2 - sensitivity analysis**  **Adjusted mean difference (I-C)**  **(N=1007)** | | | **MI - sensitivity analysis**  **Adjusted mean difference (I-C)**  **(N=2075)** | | |
| --- | --- | --- | --- | --- | --- | --- | --- | --- | --- | --- | --- |
|  | **mean (SE)** | **mean (SE)** | **estimate** | **95% CI** | **p-value** | **estimate** | **95% CI** | **p-value** | **estimate** | **95% CI** | **p-value** |
| Costs (£) |  |  |  |  |  |  |  |  |  |  |  |
| Total | 524.30 (91.75) | 528.67 (67.34) | 30.24 | -140.98 to 201.47 | 0.73 | 16.44 | -155.32 to 188.21 | 0.85 | 252.00 | -221.00 to 725.00 | 0.31 |
| Outcome |  |  |  |  |  |  |  |  |  |  |  |
| SDQ Total Difficulties score | 5.17 (0.57) | 5.39 (0.45) | -0.54 | -1.68 to 0.61 | 0.36 | -0.51 | -1.66 to 0.63 | 0.38 | -0.57 | -1.59 to 0.45 | 0.27 |

CC1 – partially adjusted complete case analysis (primary analysis); CC2 – fully adjusted complete case analysis; MI – fully adjusted analysis of imputed data

Figure 1. Bootstrapped mean differences in costs and effects of intervention compared with control (CC1)

50% of scatter points in NE quadrant

14% of scatter points in NW quadrant

29% of scatter points in SE quadrant

7% of scatter points in SW quadrant

-£400

-£200

£200

£400

*Difference in cost*

2

1

-1

-2

-3

*Difference in SDQ score*

95% confidence ellipse

More effective

Less effective

More costly

Less costly

CC1 – partially adjusted complete case analysis (primary analysis); NE=northeast; SE=southeast; SW=southwest; NW=northwest

Figure 2. Cost-effectiveness acceptability curves showing the probability that TCM is cost-effective compared to TAU for different values of willingness to pay thresholds

0%

20%

40%

60%

80%

100%

Probability TCM is cost-effective

£0

£500

£1000

£1500

£2000

Willingness to pay for unit change in SDQ Total Difficulties Score

CCI

CC2

MI

CC1 – partially adjusted complete case analysis (primary analysis); CC2 – fully adjusted complete case analysis; MI – fully adjusted analysis of imputed data

### References

**(DH), D. o. H.** (2015). NHS Reference Costs 2014–15. London DH.

**Barett, B., Byford, S., Chitsabesan, P. & Kenning, C.** (2006). Mental health provision for young offenders: service use and cost. *The British Journal of Psychiatry* **188**, 541-546.

**Barmby, P.** (2006). Improving teacher recruitment and retention: the importance of workload and pupil behaviour. *Educational Research* **48**, 247-265.

**Byford, S., Harrington, R., Torgerson, D., Kerfoot, M., Dyer, E., Harrington, V., Woodham, A., Gill, J. & McNiven, F.** (1999). Cost-effectiveness analysis of a home-based social work intervention for children and adolescents who have deliberately poisoned themselves. Results of a randomised controlled trial. *Br J Psychiatry* **174**, 56-62.

**Curtis, L.** (2015). Unit Costs of Health and Social Care. Canterbury: PSSRU.

**National Insitute for Health and Clinical Excellence** (2013). Guide to the methods of technology appraisal. NICE: London.

**Netten A., K. J., Dennett J., Cooley R., Slight A.** (1998). *A ''Ready Reckoner'' for Staff Costs in the NHS*. PSSRU, University of Kent, Canterbury, Kent.

**Office for National Statistics** (2015). Annual Survey of Hours and Earnings: 2015.

**Royal Pharmaceutical Society of Great Britain** (2016). British National Formulary. BMJ Group and RPS Publishing.
